# Clinical cardiac assessment in newborns with prenatally diagnosed intrathoracic masses

**DOI:** 10.1186/s13052-018-0543-4

**Published:** 2018-08-22

**Authors:** Ingrid Anne Mandy Schierz, Mario Giuffrè, Ettore Piro, Maria Clara Leone, Giuseppa Pinello, Giovanni Corsello

**Affiliations:** Neonatal Intensive Care Unit, AOUP “P. Giaccone”, Department of Sciences for Health Promotion and Mother and Child Care “G. D’Alessandro”, University of Palermo, Via Alfonso Giordano n. 3, 90127 Palermo, Italy

**Keywords:** Retrospective study, Respiratory system abnormalities, Congenital diaphragmatic hernia, Cardiovascular abnormalities

## Abstract

**Background:**

Congenital space-occupying thoracic malformations and diaphragmatic hernia have in common pulmonary hypoplasia. Our study aims to assess cardiac involvement during post-natal adaptation.

**Methods:**

A retrospective study was carried out on newborns with prenatally diagnosed intrathoracic mass. Gathering for respiratory distress syndrome (RDS), 35 neonates were compared for clinical course, cardiovascular enzymes, ECG, and ultrasound.

**Results:**

The analysis revealed a high left heart defect rate in patients with severe RDS, without being influenced by the laterality. Ultrasound or laboratory assessment did not detect altered cardiac dimension or cardiomyopathy. Solely ECG signs of right ventricular strain were found. Increased QT-dispersion, T-wave and cardiac variability alterations in the first hours were all expression of non-specific cardiac repolarization disorders but predict worse outcome.

**Conclusions:**

Although RDS is the predominant symptom, slight cardiac dysfunctions should be recognized for prompt treatment. Conventional examinations for postnatal adaption should be integrated by complementary investigations.

## Background

Advanced prenatal ultrasound (US) screening program allows the diagnosis of intrathoracic masses, but above all may influence follow-up, early therapeutic strategies, prognosis, and parental expectations. Rare space-occupying thoracic malformations such as congenital cystic adenomatoid malformation (CCAM; incidence 0.11‰ live births) [1], pulmonary sequestration complex, congenital lobar emphysema, bronchial atresia, foregut duplication cysts, and chest tumors, must be differentiated from displaced abdominal organs due to congenital diaphragmatic hernia (CDH; incidence 0.28‰ live births) [1]. All these conditions must be evaluated for concomitant congenital pulmonary hypoplasia (congenital small lung) [2–4].

Multiple malformations can occur in the same patient implicating a common embryopathogenesis (pulmonary sequestration associated with CDH, CCAM, and lung lobe anomalies, or eventratio and enterogenous cysts) [5–12]. The normal lung development proceeds simultaneously by the interaction of bronchial, arterial, venous, and lymphatic tree. Consequently, the symptomatology, and thus the prognosis, depends not only on the respiratory function but also on the cardiovascular and lymphatic involvement [3]. All these malformations are characterized by the risk of cardiomediastinal shift, loss of diaphragmatic thickness and concavity, nonimmune hydrops (pleural/pericardial effusion, ascites, scalp/integumentary edema, placentomegaly, Ballentyne syndrome), and cardio-pulmonary insufficiency, requiring intensive prenatal and postnatal surveillance and timely therapy [4, 7, 8, 10–17]. Mortality rate resulting from CCAM with associated early hydrops and without intervention or maternal betamethasone administration can reach up to 100% [13, 15–17]. Despite promising preliminary results of fetal medicine and advanced neonatal intensive care with aggressive treatment of pulmonary arterial hypertension, mortality from CDH remains high, not only when heart defects are associated [4, 18].

The aim of the present study was to assess cardiac involvement during postnatal pulmonary (mal-)adaptation in newborns with lung hypoplasia secondary to congenital pulmonary malformations or CDH.

## Methods

An institutional review board-approved retrospective study was carried out on all newborns with prenatally diagnosed intrathoracic masses delivered between 2009 and 2016 at the Neonatal Intensive Care Unit of University Hospital. Information on gestation at diagnosis, maternal medication, progression of the lesion during pregnancy, laterality, and the presence of other associated anomalies were recorded. Postnatal investigations for intrathoracic mass, degree of respiratory distress syndrome (RDS), surfactant replacement therapy, the best oxygenation index, and cardiovascular assessment during pulmonary adaption were evaluated including analyses of cardiac serum enzymes, ECG, and cardiac US indexed to Haycock-body surface area and Z-scores [19, 20]. The ECG was further analyzed for QRS wave amplitudes, Sokolow-Lyon-index (RV_6_ + SV_1_), Cornell (RaVL+SV_3_) criterion [21]; heart rate (RR- or NN-interval) variability, standard deviation of NN intervals, number of adjacent NN intervals differing by more than 50 ms, rate of NN50 [22]; QTc and QT dispersion [23]. For the comparison of the cardiac data, the patient group was paired with a control group of newborns with the same gestational age and without RDS enrolled in a previous study. Parent’s informed written consent was provided.

Outcome data regarding oxygenation index, days of ventilation, days of hospitalization and mortality were recorded. Genetic counseling, karyotype, additional cytogenetic and molecular analyses were performed in all cases with suspected genetic background.

Statistical analyses were performed by the open source statistical R 3.04.0 software (R Development Core Team, Vienna, Austria), and the significance was defined as *p*-value < 0.05. In a second step, the patient group was further divided by RDS gravity (absent-mild-severe), which was considered a sign of congenital pulmonary hypoplasia. RDS was defined as severe if the oxygenation index remained > 13 and the extubation was not achieved within 96 h of life, or within 96 h after neonatal surgery, or if the baby died. Baseline characteristics of the RDS groups were compared using Kruskal–Wallis tests for continuous variables, and Fisher’s exact tests for categorical variables. Multivariate analysis was performed.

## Results

Prenatal and postnatal diagnosis of fetal intrathoracic masses coincided in about 94% (33/35) of the cases. CDH with displaced abdominal organs in the thorax was the most frequent malformation (17/35; 48.5%), followed by CCAM (14/35; 40%). Patients with sequestration and anomalous pulmonary venous return (2/35; 5.7%), and postnatal discordant diagnoses such as eventratio (1/35; 2.9%) and pericardic cyst (1/35; 2.9%), were excluded for the further analysis. Perinatal characteristics were not significantly different in the malformation groups, but they differed in the RDS severity-gathering (Table 1).

**Table 1:** Perinatal characteristics of newborns with prenatally diagnosed intrathoracic masses categorized by RDS

	Controls (*n* = 31)	Without RDS (*n* = 9)	Mild RDS (*n* = 6)	Severe RDS (*n* = 16)	*p*
laterality R/L		4/5	3/3	3/13	0.27
CDH (%)	0	0	50	87.5	**< 0.0001**
mediastinal shift (%)		33.3	50	100	**0.00036**
polydramnios (%)	0	12.5	0	43.7	**0.00035**
prenatal steroids (%)	0	11	0	25	0.23
birth gestational age, weeks (range)	38^+ 6^ (29^+ 6^–40)	39 (33^+ 4^–40)	39^+ 2^ (38^+ 4^–39^+ 5^)	38^+3^ (29^+3^–39)	0.064
birth weight (g)	3200	3000	3440	2665	**0.002**
weight centile	44	37	71	16	**0.024**
SGA (%)	0	11	0	31	**0.0066**
minimum serum calcium (mmol/L)	2.4	2.45	2.25	2.3	0.38
CK (μkat/L)	3.6	10	9	5.5	0.019
Apgar score 1′/5’	9/10	9/10	8/9	3/7	**< 0.0001**
days of hospitalization	3	6	20	25	**< 0.0001**
mortality (%)	0	0	0	37.5	**< 0.0001**

*Abbreviations*: *RDS* respiratory distress syndrome, *CDH* congenital diaphragmatic hernia, *CK* creatine kinase, *SGA* small for gestational age

Significant differences are in bold

Mediastinal shift, polydramnios, and intrauterine growth restriction (IUGR) were correlated with more severe RDS and poor neonatal outcome. Serum cardiac enzymes, surfactant replacement therapy, or the best oxygenation index from 1st day were not correlated with outcome. Associated extracardiac malformations were found in 9/16 patients with severe RDS (*p* = .00016).

The cardiovascular assessment revealed a high rate of congenital heart defects (CHD) in patients with severe RDS. In this group of patients, aortic and ductal arch anomalies and ventricular septal defects (VSD) were detected. In one infant VSD was associated with Edwards syndrome, and in one with 22q11.2 deletion syndrome. The laterality of the mass was not statistically associated with cardiac assessment or CHD rate.

The Sokolow index was significantly augmented in sequestration complex group but it was normal in newborns affected by CDH or CCAM, with or without RDS. Differences in cardiac US and ECG variables in the RDS subgroups were reported in Table 2.

**Table 2:** Medians of characteristic clinical cardiac parameters from newborns with prenatally diagnosed intrathoracic masses, categorized by RDS

	Controls (*n* = 31)	Without RDS (*n* = 9)	Mild RDS (*n* = 6)	Severe RDS (*n* = 16)	*p*
CHD (%)	0	11.5	0	31	**0.005**
left ventricular cardiac index (L/min/m^2^)	3.45	3.4	N/A	2.9	0.47
left ventricular diastolic diameter (Z Score)	−0.49	−0.79	N/A	−1.75	0.28
aortic valve diameter (Z score)	0.42	1.71	−0.03	0.52	0.35
pulmonary valve diameter (Z score)	−0.46	−1.49	−1.91	−0.15	0.088
QRS axis	121	91.5	105	83.5	0.13
Sokolow index	1.2	1.032	0.957	1.15	0.31
Cornell index	0.8	0.75	1.025	0.2	0.098
amplitude R/S V2	1.14	2.1	0.4	0.75	**0.0025**
amplitude RV1 (mV)	1.1	0.7	0.4	0.5	**0.002**
amplitude RV6 (mV)	0.5	0.375	0.3	0.15	**0.04**
amplitude SV6 (mV)	0.35	0.025	0.4	0.2	**0.015**
QT dispersion (ms)	60	60	70	100	**0.032**
QTc dispersion (ms)	82	84	104	137.5	**0.01**
precordial T-wave inversion	3	2.5	1	2	**0.023**
SDNN (ms)	29.23	28.59	11.48	9.25	**0.001**

*Abbreviation*: *RDS* respiratory distress syndrome, *CHD* congenital heart defects, *N/A* not available

Significant differences are in bold

## Discussion

The clinical presentation of infants with pulmonary hypoplasia depends on the severity of the disease and the underlying cause. In severe forms, RDS, hypercapnia, and hypoxemia consistently occur with signs and symptoms of associated conditions.

We investigated the cardiac involvement in newborns with pulmonary hypoplasia secondary to intrathoracic space-occupying lesions. In newborn, the suspected cardiac impact derived from the intrathoracic mass effect can be explained by almost three physiopathological mechanisms.

In fetal echocardiograms it has been demonstrated that an increased intrathoracic pressure in hydropic fetuses depresses global fetal growth, decreases left ventricular and aortic dimensions, and tends to increase the inferior vena cava dimension, reflecting the inability of the heart to adequately drain the venous system [24–26]. However, cardiac dimension in our study population was within the normal range and did not differ significantly between groups. In all neonates, during the first few hours of cardiopulmonary transition, the optimal left ventricular filling was not achieved, and this could have resulted in a diminished global left ventricle dimension. Another explanation could be that the real ventricular diameters in the RDS groups might be overestimated due to not always optimal insonation due to mediastinal shift. The literature refers to significant reduced diameters only in cases with hydrops, which were not observed in our cases.

The concept of left-sided cardiac underdevelopment could be highlighted also by the increased frequency of left-sided CHD (11–29%) in the CDH population, ranging from severe hypoplastic left heart syndrome to asymptomatic bicuspid aortic valve or VSD [18, 25–28]. We can confirm this high prevalence of left CHD, but without the influence of the laterality of the malformation. CCAM usually occurs as a sporadic nonhereditary disease, rarely accompanied by other anomalies, predominantly renal anomalies/tumors, VSD (10%) [8], conotruncal CHD (1.9%) [29], and ECG abnormalities, attributable to associated autosomal chromosome trisomies or 17p deletion syndromes (Hornstein-Birt-Hogg-Dubé or Smith-Magenis syndrome) [7, 12, 29, 30]. Candidate genes like *HOXB5* located in chromosome 17p11.2 and *FGF7* located in chromosome 15, actuating transcription factors and growth factors, seem to be involved in this disrupted epithelio-mesenchymal crosstalk [29]. In a Notch signaling inhibited mouse-model (the Pax3Cre/+ mouse), the development of both the cardiac neural crest derivatives (developing aortic arch vessels and the conotruncal septum) and the derivatives of the somites, including skeletal muscle of the diaphragm and limbs, were altered, but surprisingly the major cause of death at birth in these mice was respiratory failure, and not heart failure [31]. Given that it is probable that a genetic entity is characterized by CCAM (or CDH) and CHD [29, 31], the still unknown interaction of altered gene as a primary event prevail over the secondary hydrops effect theory of left-sided cardiac underdevelopment.

The second physiopathological aspect of the intrathoracic mass effect derives from the degree of lung hypoplasia and sustained pulmonary arterial hypertension owing to decreased pulmonary arterial growth and hypertensive vascular remodeling. Consequently, there are findings of right hypertrophic cardiomyopathy and intracardiac and extracardiac right-to-left shunts across a patent ductus arteriosus, a patent foramen ovale, and/or intrapulmonary anastomotic vessels [32]. However, hypertrophic cardiomyopathy in the neonatal age must be distinguished from other causes as CHD, asphyxia, infections, supraventricular tachycardia, diabetic cardiomyopathy, or large extrapulmonary arterio-venous malformations.

Finally, the presence of the above mentioned intrapulmonary shunts (seen in CCAM, sequestration, CDH as well as in alveolar capillary dysplasia or bronchopulmonary dysplasia) [32] suggests a potential mechanism of refractory hypoxemia with further hemodynamic impact on left ventricle dysfunction and the risk of congestive heart failure [10, 33–35]. The regression of cardiac symptoms after surgical treatment of these pulmonary malformations has been reported [34–36]. Nevertheless, also isolated lung hypoplasia without associated malformations has been reported with dilated cardiomyopathy [37].

Even though we also enrolled newborns with severe RDS and hypoxemia who died during the first day of life before surgery, we could not find evidence of significant signs of cardiomyopathy by US or laboratory cardiac assessment. Solely ECG signs of right ventricular strain were detected in patients with severe RDS. Only in patients with pulmonary sequestration we observed a further correlation between right heart impairment and spectral-Doppler US. Probably, the lack of correlation in the other patient groups could be attributed both to small study groups and the timing of cardiological exam soon after admission. It would take time to develop important postnatal intrapulmonary shunts and cardiomyopathy. Due to the study design, we could not retrospectively measure more specific ECG or US parameters for suspected concomitant diastolic impairment, like Tei-index or tissue Doppler.

For the first time, in the severe RDS group we could find evidence of significant early non-specific signs of cardiomyopathy and cardiac repolarization disorders like increased QT-dispersion, T-wave alterations, and reduced cardiac variability by ECG. Although non-specific and still not detectable by US or laboratory investigations, they can anticipate worse outcome when detected in the first few hours. In the multivariate regression analyses these anomalies were independent from gestational age. Significant spatial heterogeneity of ventricular repolarization in newborns with severe aortic coarctation [38] or with IUGR [23], due to relative interventricular septum hypertrophy and electrophysiological remodeling of the heart, is reported. Both rates of IUGR and left-sided CHD are high among newborns with intrathoracic masses, also in our study, therefore it is not clear if heterogeneity of ventricular repolarization is ascribable to these variables or is due to hypoxemic severe RDS. Data from histology and cellular immunophenotyping support the cardiovascular remodeling hypothesis which consists of an inhomogeneous distribution of ventricular growth factors and muscle proteins [39]. Subtle ECG repolarization disorders could be attributed to these non-homogenous cardiac muscle alterations.

Previous reports have suggested that the combination of CDH and CHD is a strong predictor of prenatal and postnatal mortality [40], even in the presence of cardiac lesions (e.g. VSD) that generally have excellent survival rates and do not cause significant hemodynamic compromise in neonatal age [27, 28]. Apart from CHD, also prenatal lung volume estimate, fetal MRI lung-to-liver signal intensity ratio, liver herniation, premature birth, or best oxygenation index on day 1 of life as lung function markers, but not prenatal Z-scores of left heart structures, have been reported as significant independent predictors of mortality in CDH [28, 41]. Fetal MRI lung volume measurements are the most useful predictor of perinatal outcomes also in fetuses with CCAM, as they best reflect the severity of pulmonary hypoplasia [42, 43].

## Conclusions

Although in the early neonatal age RDS is the predominant symptom in all intrathoracic space-occupying lesions, a subtle early cardiac dysfunction must be recognized for prompt treatment before irreversible cardiac damage occurs. Conventional US for postnatal pulmonary and cardiac evaluation should be integrated by complementary dynamic, non-invasive, pain-free and ionizing radiation-free investigations.

## Abbreviations

CCAM: Congenital cystic adenomatoid malformation
CDH: Congenital diaphragmatic hernia
CHD: Congenital heart defects
IUGR: Intrauterine growth restriction
RDS: Respiratory distress syndrome
US: Ultrasound
VSD: Ventricular septal defects
